# A radiological evaluation of alveolar bone regeneration between the left and right mandibles and maxillae of the Chacma baboon

**DOI:** 10.4102/jsava.v87i1.1310

**Published:** 2016-09-06

**Authors:** Marthinus J. Kotze, Kurt-W. Bütow, Steve A. Olorunju, Harry F. Kotze

**Affiliations:** 1 Department Maxillo-Facial and Oral Surgery, University of Pretoria, South Africa; 2 College of Health Sciences, University of KwaZulu-Natal, South Africa; 3 Medical Research Council of South Africa, Pretoria, South Africa; 4 Department of Cardiothoracic Surgery, University of the Free State, South Africa

## Abstract

There is a lack of information in comparing the healing rate between the left and right sides of the maxilla and mandible. Osteogenesis of alveolar bone was evaluated with digital radiology by comparing differences in bone density (BD) at different time points within the left and right maxilla and mandible. Alveolar bone defects were created in five healthy Chacma baboons. Standardised x-ray images were acquired over time and the densities of the selected trauma areas were measured pre-operatively, post-operatively and at 3 and 6 weeks post-operatively. Differences in densities were statistically tested. There was no significant difference when the grey scale averages of the combined first and fourth quadrants (right side) and combined second and third quadrants (left side) were compared pre-operatively (*t* = 0.70), immediately post-operatively (*t* = 0.34), 3 weeks post-operatively (*t* = 0.40) and 6 weeks post-operatively (*t* = 0.66). There was also no significant difference between the values for the first and second quadrants (maxilla) pre-operatively (*t* = 0.37), immediately post-operatively (*t* = 0.30), 3 weeks post-operatively (*t* = 0.30) and 6 weeks post-operatively (*t* = 0.38); the third and fourth quadrants (mandible) were also not significantly different pre-operatively (*t* = 0.29), immediately post-operatively (*t* = 0.69), 3 weeks post-operatively (*t* = 0.07) and 6 weeks post-operatively (*t* = 0.06). However, the results showed an increased predisposition of the right side to regenerate faster than the left side and indicated sufficient information to investigate the effect of laterality and preferred side of mastication on the rate of healing and alveolar BD in the maxilla and mandible.

## Introduction

Animal models are often used in dentistry as biological models in research projects on alveolar bone healing and regeneration, especially in periodontology and implantology. Dogs and monkeys have most often been used in periodontal research (Dannan & Alkattan 2007). If a possibility existed of obtaining block biopsies, baboons, rhesus monkeys and cynomolgus monkeys have been used to study osseo-integrated oral implants (Blumenthal *et al*. 2003).

Dental implants, as used in various disciplines of dentistry, have changed the face of dentistry over the past 40 years. As with most treatment procedures in dentistry today, dental implants not only involve scientific discovery, research and understanding but also application in clinical practice. One of the main barriers to a successful dental implant procedure is lack of bone quality and therefore, bone density (BD). According to definition (United States National Library of Medicine [NLM]), BD is the amount of bone tissue in a certain volume of bone. Common modalities, amongst others, used for determining BD are dual-energy X-ray absorptiometry (DEXA) (Corten *et al*. 1993) and quantitative computed tomography (QCT) (Adams 2009). However, these methods are expensive and time consuming. Very accurate BD measurement is feasible using plain digital radiography (Bittar-Cortez *et al*. 2006; Kinds *et al*. 2011; Rosholm *et al*. 2001). With digital radiography, an inexpensive and uncomplicated method was demonstrated to evaluate bone regeneration after trauma and to follow the process over a period of time (Kotze, Bütow & Kotze 2012).

Masticatory forces, the hardness of food eaten and the instigated strain patterns have an influence on symphyseal fusion, bone formation and development of the mandible in primates (Hylander 1979; Ravosa 1996). The loading history of bones and energy transfer concepts can be applied to many different situations of growth, functional adaptation, injury and ageing of connective tissues (Carter, Fyhrie & Whalen 1987). In a human model, the overall mandibular BD distribution results in a tubular structure that is known to provide the bone with increased strength and to resist bending and torsion during mastication (Chou *et al*. 2015). The development of dense bone at the genion region is affected by the mandibular flexure during functioning. A reduced loading on the bone due to various types of edentulism in the mandible results in clinically observed BD change patterns with the greatest bone loss in total edentulous mandibles (Chou *et al*. 2015). A difference in the forces and strain between the working and balancing sides is described (Hylander 1979), not only in the muscle insertion area but also in mandibular alveolar bone in the molar region (Kawamura *et al*. 2005). It has been demonstrated that there is a significant difference in the alveolar BD between the maxilla and mandible at different sites (Kotze *et al*. 2014; Park *et al*. 2008). Therefore, it is anticipated that there should be a difference of BD in the rate of healing following trauma between the left and right sides of the mandible and maxilla due to different muscle actions and strains. No literature could be found comparing the left and right sides of the alveolar BD in healthy bone or the rate of alveolar bone healing following trauma.

The aim is to evaluate BD radiologically, as well as the rate of healing of alveolar bone from the left and the right sides of the mandible and maxilla of the Chacma baboon over a period of 6 weeks.

## Materials and methods

### Subjects and ethical considerations

Five healthy Chacma baboons of similar sex, age and weight were used and numbered 1–5. Approval for the study was granted by both the Animal Use and Care Committee (AUCC) and a subcommittee of the Committee for Research Ethics and Integrity at the University of Pretoria and North West University (Kotze *et al*. 2014). The animals were anaesthetised with ketamine hydrochloride (10 mg/kg), and haemostasis and pain were controlled with 1.8 mL (9 mg) bupivacaine with 0.5% epinephrine 1:200 000 (as bitartrate) (Novocol, Pharmaceuticals of Canada. Inc., Cambridge, Ontario, Canada) (Kotze *et al*. 2014).

### Surgical procedure

**FIGURE 1:**
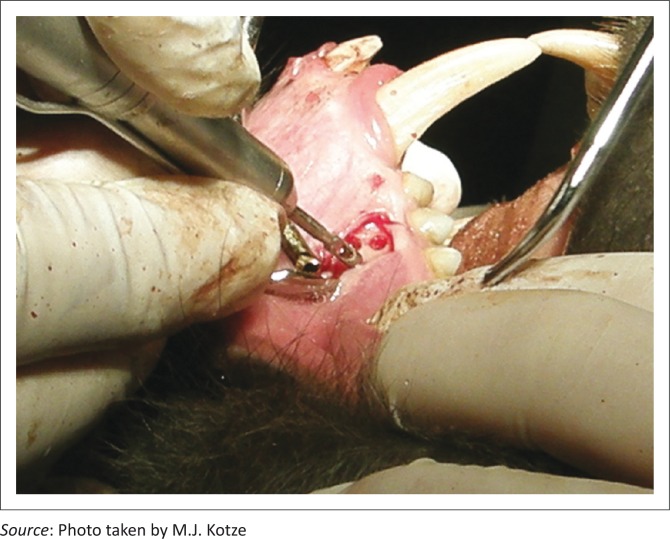
Three bone defects were positioned at 2 mm from each other.

**FIGURE 2:**
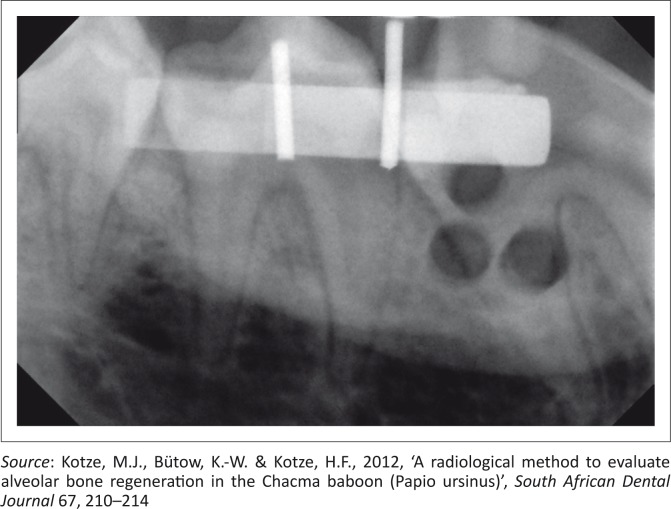
Directly post-operatively radiograph of right mandible with the three bony defects visible.

With a trephine burr fitted to a straight hand piece and a surgical unit, three similar bone defects per quadrant (Quadrant 1 [Q1] being the right maxilla, Q2 the left maxilla, Q3 the left mandible and Q4 the right maxilla) were created in the premolar areas of the respective bones (Figures 1 and 2). The alveolar bone defects were 3 mm deep and 2 mm apart from each other and angled at 90 degrees to the surface of the alveolar bone (Kotze *et al*. 2012).

### Radiology

**FIGURE 3:**
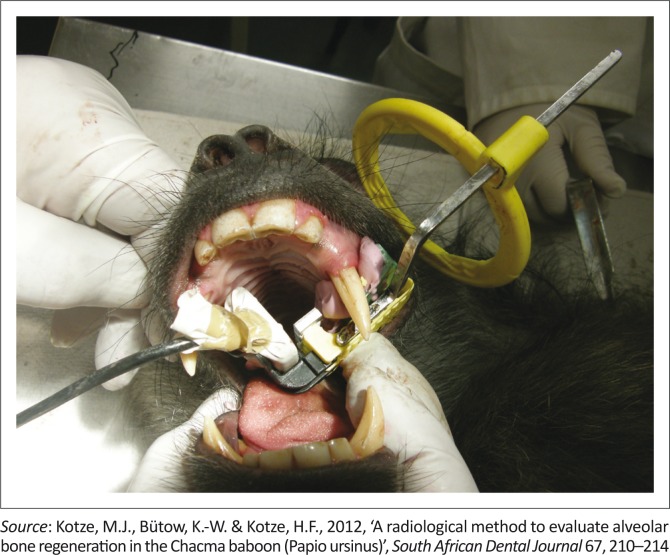
An image of the positioning of the apparatus in the mouth of the baboon before an image was acquired.

**FIGURE 4:**
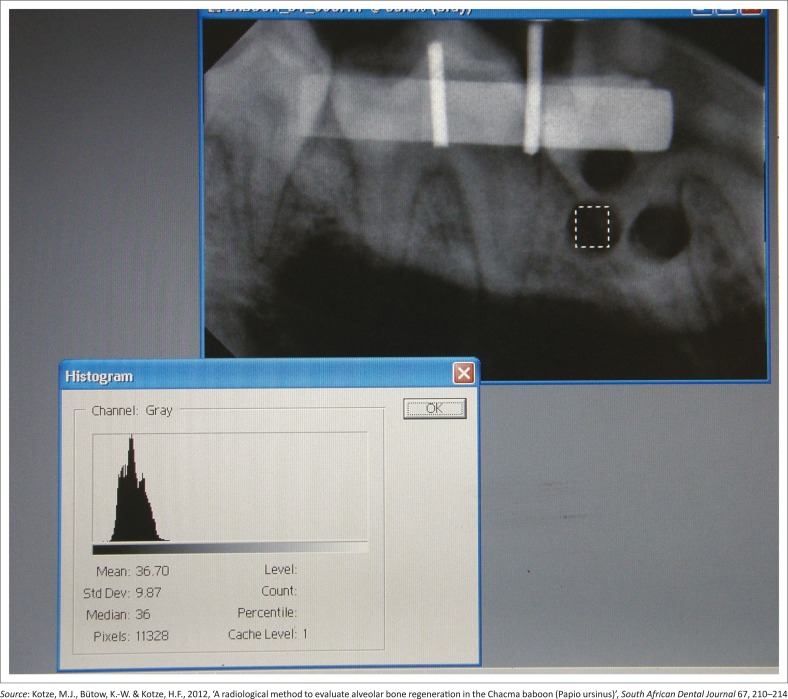
An image of the computer screen displaying the histogram and reflecting the average radiographic intensity of the image of the selected area of the created defect.

Standardised reproducible radiographs (Figure 2) were taken in the premolar area at each of the four quadrants pre-operatively, directly post-operatively and again after 3 and 6 weeks. To enable reproducible radiographs, a device consisting of a sectional impression tray with impression putty, a bite block (XPC-DS Digital Position System, Gendex, Lake Zurich, Illinois), a sensor (Gendex Visualix EHD Digital intra-Oral x-ray unit – Size 1 with 25.6 line pairs per mm) and a custom made aluminium step wedge (Figure 3) was used. The step wedge was custom made due to the limited space in the mouth of the baboon (Figure 4). Four digital images were acquired per quadrant at the predetermined time intervals. Therefore, records were taken of 16 images per animal at each of the four time periods. The method used for the acquisition of the standardised radiographs was described in detail by Kotze *et al*. (2012).

### Evaluation methods

The images acquired for each quadrant were imported into Adobe Photoshop (V6.0; Adobe Systems Inc., San Jose, CA, USA). Using the histogram, contrast and brightness tools of the Adobe Photoshop software, average grey scale values of the selected regions of interest (ROI) on the image of the aluminium step wedge were standardised across all four images for each quadrant. A transparent sheet was placed over the screen and the ROI was marked on the sheet, so that it was possible to accurately position the radiograph in the exact position with repeated evaluations. An ROI was also selected on each of the defect sites on each of the images taken immediately post-operatively. The average grey scale values for these areas on each of the three defects on each image were determined and recorded. This was done for all images of a quadrant acquired from each of the different time periods (Figure 4). Only one blinded examiner made the measurements and recorded the results. The analysis of the data was standardised and repeatable, which implies that there was no reason to measure inter-observer variation (Kotze *et al*. 2014). The method used was described by Kotze *et al*. (2012).

### Statistical analyses

Analysis of variance (ANOVA) for repeated measures (analysis of variance considering more than one period) was used to test for possible differences in the data set. Periodic changes (actual and percentage) were analysed using the two-sample *t*-test within each time point to compare the left and right sides of the mandible and maxilla (Kotze *et al*. 2014).

## Results

With the use of the ANOVA for repeated measures, the average grey scale values from pre-operatively, immediately post-operatively, 3 weeks post-operatively and 6 weeks post-operatively for the four quadrants from each animal were compared. The results are summarised in Table 1. There was no significant difference when the grey scale averages of the combined first and fourth quadrants (right side) and combined second and third quadrants (left side) were compared for pre-operatively (*t* = 0.70), immediately post-operatively (*t* = 0.34), 3 weeks post-operatively (*t* = 0.40) and 6 weeks post-operatively (*t* = 0.66). The combined results for comparison of the average grey scale values for all the animals between the first and second quadrant are summarised in Table 2. There was also no significant difference between the values for pre-operatively (*t* = 0.37), immediately post-operatively (*t* = 0.30), 3 weeks post-operatively (*t* = 0.30) and 6 weeks post-operatively (*t* = 0.38). When the third and fourth quadrants were compared for all the animals (Table 3), there was no significant differences in pre-operatively (*t* = 0.29), immediately post-operatively (*t* = 0.69), 3 weeks post-operatively (*t* = 0.07) and 6 weeks post-operatively (*t* = 0.06). For animal 3, significant differences were noted between quadrant 3 and quadrant 4 between baseline (pre-operatively) and immediately post-operatively (*p* = 0.003) on the one hand, and 3 weeks post–operatively (*p* = 0.005) and 6 weeks post-operatively (*p* = 0.010) on the other hand.

**TABLE 1:** Average grey scale values for the combined left and right maxillae and mandibles for each animal.

Animal	Side	Pre-op	Post-op	3 weeks	6 weeks
1	R	91.6	46.2	50.9	59
	L	103.2	70.6	76.7	79.3
2	R	103.5	49.4	53	59.2
	L	83.2	45.4	49.5	57
3	R	99.4	36.2	46.1	49.5
	L	57.2	10.1	10.9	13.5
4	R	60.7	29.4	34.1	45.1
	L	98.1	40.2	43	49
5	R	60.7	30.1	35	40.9
	L	91.6	60.3	70.4	72.7

R, quadrants 1 and 4; L, quadrants 2 and 3; Pre-op, pre-operatively; Post-op, immediately post-operatively; 3 weeks, 3 weeks post-operatively; 6 weeks, 6 weeks post-operatively.

**TABLE 2:** Average grey scale values for the left and right maxilla of each animal.

Animal	Side	Pre-op	Post-op	3 weeks	6 weeks
1	Q1	111.8	52.6	58	69.3
	Q2	120.6	69.1	73.85	76.7
2	Q1	102.1	32.1	35.2	44.3
	Q2	116.9	71.5	75.6	89.3
3	Q1	99.3	32.5	38	41.9
	Q2	40.4	3.6	4.3	5
4	Q1	84.2	45.5	49.2	67
	Q2	124.6	54.9	59.3	61.4
5	Q1	73.9	35.5	43.7	52.3
	Q2	108.4	86.9	8.3	88.4

Q1, quadrant 1; Q2, quadrant 2; Pre-op, pre-operatively; Post-op, immediately post-operatively; 3 weeks, 3 weeks post-operatively; 6 weeks, 6 weeks post-operatively.

**TABLE 3:** Average grey scale values for the left and right mandibles of each animal.

Animal	Side	Pre-op	Post-op	3 weeks	6 weeks
1	Q3	85.7	72.1	79.5	81.9
	Q4	71.5	39.8	43.7	48.8
2	Q3	49.7	19.2	23.5	24.8
	Q4	105	66	70.8	74.2
3	Q3	74.2	16.5	17.5	22
	Q4	99.5	39.8	54.3	57.2
4	Q3	71.8	25.5	26.8	36.6
	Q4	37.2	13.2	19.1	23.2
5	Q3	74.9	33.6	52.5	57
	Q4	47.5	24.8	26.4	29.6

Q3, quadrant 3; Q4, quadrant 4; Pre-op, pre-operatively; Post-op, immediately post-operatively; 3 weeks, 3 weeks post-operatively; 6 weeks, 6 weeks post-operatively.

When the group of animals was compared (Table 4), it was not surprising that the pre-operative density was not significantly different between the left and right sides of the maxilla and mandible (*t* = 0.70), as similar areas were investigated on both sides. Furthermore, because a similar quantity of bone tissue was removed from all the defect areas, no significant difference was found immediately post-operatively (*t* = 0.34) between the left and right sides of the mandible and maxilla.

**TABLE 4:** Average grey scale values for the combined maxillae and mandibles of all the animals.

Side	Pre-op	Post-op	3 weeks	6 weeks
R	83.2	38.2	43.8	50.8
L	86.7	45.3	50.1	54.3
*t*-value	0.7	0.34	0.4	0.66

R, quadrants 1 and 4; L, quadrants 2 and 3; Pre-op, Pre-operatively; Post-op, immediately post-operatively; 3 weeks, 3 weeks post-operatively; 6 weeks, 6 weeks post-operatively.

**FIGURE 5:**
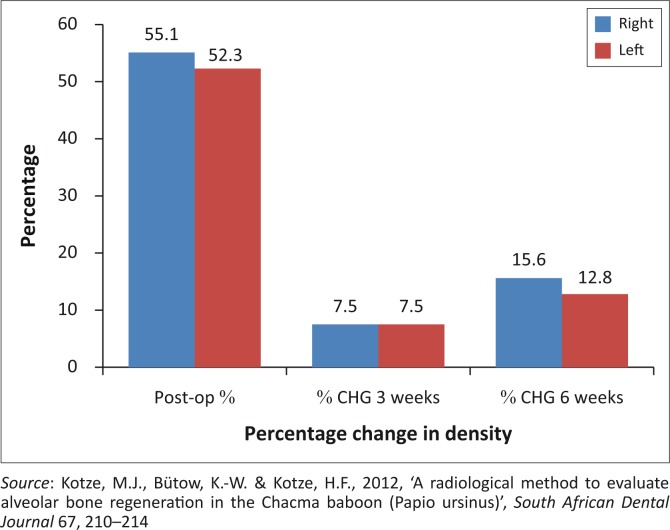
Percentage change in density after 3 and 6 weeks.

The healing process of the defect areas was similar without any significant differences between the left and right sides after 3 weeks post-operatively (*t* = 0.040) (*p* > 0.05) and 6 weeks post-operatively (*t* = 0.66) (*p* > 0.05). The rate of healing or the change in density for the first 3 weeks (Figure 5) was similar for the left and right sides. However, the change in density and healing from 3 weeks post-operatively to 6 weeks post-operatively was higher (2.8%) in the right mandible and maxilla, although not significantly so (Figure 5).

## Discussion

Bone density and bone regeneration were investigated in the premolar area of the maxilla and mandible in similar areas. Change in density, which is reflected in a change in average grey scale value (AGV), was derived from radiographs in the region of interest. The radiological changes in BD were previously compared with histological and histomorphometrical measurements (Cakıcı *et al*. 2011; Gulsahi *et al*. 2010) as well as with digital radiography (Kotze *et al*. 2012), and a correlation was found between the different approaches. Therefore, the principle of change in radiological density over time can be used as an indicator of bone turnover.

The premolar area in baboons represents the more dense areas in the mandible and maxilla as the density showed a progressive increase from the incisor to the retro molar area (Chugh *et al*. 2013; Park *et al*. 2008). Baboon and other non-human primate bones exhibited similar fracture, microstructural and compositional properties to those of humans, compared with other animals such as cats, rodents, rabbits, guinea pigs and mini pigs, sheep, goats and dogs (Turner 2001; Wang, Mabrey & Agrawal 1998).

This increased BD in the premolar area of maxilla and mandible is probably due to more masticatory forces in these areas. Laterality, hand dominance and preferred side to chew may also have an influence on the difference in BD. A positive correlation, with the right hand more dominant, was found between handiness and laterality index in free-ranging bonnet macaque monkeys whilst filling and emptying their cheek pouches (Mangalam, Desai & Singh 2014). No significant difference was found when the left and right sides of the maxilla (*p* > 0.05) and left and right sides of the mandible (*p* > 0.05) were compared. However, the laterality, hand dominance and preferred side to chew were not investigated in this study. One animal revealed a difference in BD between the left and right mandibles. A previously published study presented results that alveolar bone remodelling is activated by the flexure produced by mechanical stimulation from mastication through the periodontal membrane (Sato *et al*. 2005). It is also suggested the cortical bone might increase in density and thicken as masticatory function develops (Sato *et al*. 2005). The mandible, which is the only moving bone section of the skull, is moved by the muscles of mastication, which are responsible for a variety of three-dimensional movements in the coronal, sagittal and transverse planes. Each movement produces a force or combination of forces transferred through the teeth to the alveolar bone of the mandible and maxilla. It could be possible that this specific animal masticated primarily on the right side, whilst the others were laterality neutral thus, equally left and right, as found in the macaque monkeys (Mangalam *et al*. 2014). The animals used in the present study were on a natural diet specific for the species. The Chacma baboon is an omnivore with different hardness of food (Hamilton, Buskirk & Buskirk 1978). The molars of the Chacma baboon are positioned relatively far forward to the temporomandibular joint, which results in the baboon being able to exert relatively greater muscle forces during posterior biting in comparison to humans (Mavropoulos *et al*. 2004). The average weight (19.8 kg ± 4.3 kg) of the animals implied young animals, as the weight of an adult male Chacma baboon can reach up to 40 kg in 5 years (Estes 1992). Therefore, it is presumed that the animals were still in their developing phase, with immature alveolar bone, and possessed an active osteogenic environment that resulted in faster bone healing (Lindaman 2001; Loh, Lee & Yeap 1988).

## Conclusion

Radiography made evaluation of BD and healing of iatrogenic bone defects in the premolar areas of both mandibles and maxillae over a 6-week time period possible. Although no significant difference between the left and right sides of the maxilla and mandible was found in alveolar BD and regeneration over 6 weeks, the results showed an increased tendency of the right side to regenerate faster than the left side. Irrespective of the small sample size and utilisation of only the premolar area as the area of interest, the results provide sufficient information for future investigation of the effects of laterality and preferred side of mastication on the rate of healing and alveolar BD in the maxilla and mandible.
